# A systematic review and meta-analysis of the prevalence and risk factors of type 2 diabetes mellitus in Nigeria

**DOI:** 10.1186/s40842-024-00209-1

**Published:** 2024-12-06

**Authors:** Michael Adeyemi Olamoyegun, Kehinde Alare, Samson Adedeji Afolabi, Nicholas Aderinto, Taiwo Adeyemi

**Affiliations:** 1 Department of Medicine, Endocrinology, Diabetes & Metabolism (EDM) Unit, Ladoke Akintola University of Technology/LAUTECH Teaching Hospital, Ogbomoso, Oyo State Nigeria; 2 Department of Medicine, Ladoke Akintola University of Technology, Ogbomoso, Nigeria

**Keywords:** Type 2 diabetes, Prevalence, Risk factors, Review, Nigeria

## Abstract

**Background:**

Type 2 diabetes mellitus (T2DM) is a major global non-communicable disease, leading to increased morbidity and mortality. Its prevalence in Nigeria is driven by various risk factors. This review assesses the national and regional prevalence and risk factors of T2DM in Nigeria.

**Methods:**

Following PRISMA guidelines, electronic databases (PubMed, Scopus, Google Scholar, African Journals Online) and gray literature were searched for English-language studies. The quality of the included studies was assessed using the Newcastle–Ottawa Scale. Data were extracted with Microsoft Excel and analyzed using Stata version 16 software. Random effect meta-regression analysis at 95% CI was used to assess pooled prevalence and risk factors. Heterogeneity was determined using the I^2^ statistic, and publication bias was evaluated with a funnel plot.

**Results:**

Sixty studies from different Nigerian geopolitical zones met eligibility criteria, with a total sample size of 124,876 participants and a mean age of 48 ± 9.8 years. The pooled prevalence of T2DM in Nigeria was 7.0% (95% CI: 5.0-9.0%). Moderate publication bias was observed. The South-south zone had the highest prevalence at 11.35% (95% CI: 4.52-20.72%), while the North-central zone had the lowest at 2.03% (95% CI: 1.09-3.40%). Significant risk factors included family history (9.73), high socioeconomic status (6.72), physical inactivity (5.92), urban living (4.79), BMI > 25/m^2^ (3.07), infrequent vegetable consumption (2.68), and abdominal obesity (1.81).

**Conclusion:**

The prevalence of T2DM in Nigeria (7.0%) nearly doubled the 2019 International Diabetes Federation estimate (3.7%) and shows a 21.3% increase from the 2019 review. Efforts should focus on modifying identified risk factors to reduce prevalence and prevent complications.

**Supplementary Information:**

The online version contains supplementary material available at 10.1186/s40842-024-00209-1.

## Introduction

Diabetes mellitus is one of the non-communicable diseases that contributes to the global burden of disease [1]. DM is associated with many complications including serious life-threatening micro-and macro-vascular complications resulting in increased health care costs, reduced quality of life, and increased morbidity and mortality. According to data obtained from 138 countries, the International Diabetes Federation (IDF) estimated that the global prevalence of diabetes in high-, middle-, and low-income nations was 10.4%, 9.5%, and 4.0%, respectively [2]. The IDF has estimated that 463 million adults live with diabetes worldwide (9.3%) in 2019, with a projected increase to about 800 million (10.9%) by 2045 [3]. Seventy-nine percent (79%) of those with diabetes live in low- and middle-income countries (LMICs). It is projected that diabetes cases will increase by 149% in sub-Saharan African countries in the next two decades, from 19 million in 2019 to 26 million by 2045 [4].

In Nigeria, 3.9 million adults lived with diabetes in 2019 and projected to be almost double (6.0million) by 2045 [4]. Studies, including a national survey, a systematic review and meta-analysis, community-based and other epidemiological reports, showed that the prevalence of diabetes among adults had increased substantially in Nigeria, from ~ 2.2% in 1992 to ~ 10.5% in 2022 [5–8].

In Nigeria, type 2 diabetes mellitus (T2DM) is a major health concern that is influenced by several risk factors [9, 10]. The nation's rapid urbanization, which promotes more sedentary lives and poor eating habits, is one of the main risk factors. The risk of T2DM is greatly increased by the increased use of processed foods, sugary drinks, and high-calorie meals among Nigerians who migrate to cities. This issue is further worsened by the transition from traditional diets high in fruits, vegetables, and whole grains to more Westernized diets that are low in nutrients [9–12]. The increased prevalence of obesity in Nigeria, especially among adults, is a significant risk factor for T2DM [13, 14]. The prevalence of obesity in the nation has been steadily increasing as a result of bad eating habits, restricted access to physical activity especially in cities, and a lack of knowledge about the dangers of obesity. This obesity particularly abdominal obesity impedes insulin action and results in insulin resistance [15–17]. The overall risk of developing T2DM in Nigeria is elevated by genetic predisposition in conjunction with environmental variables such as obesity, unhealthy diet, and urbanization. Therefore, to reduce the prevalence of T2DM in the nation, these risk factors must be addressed through public health initiatives such as encouraging healthy lifestyles and expanding access to healthcare. However, only a systematic review or meta-analysis has evaluated these risk factors.

Nigeria, the most populous country in Africa, has no known nation-wide surveys or any reported attempt within Nigeria in recent times to specifically determine the burden of diabetes in the country. The last national survey of non-communicable diseases (NCDs) which was conducted in 1997 put the prevalence of diabetes at 2.2% [8]. The IDF 2019 reports specifically noted that Nigeria was among countries without up-to-date data on diabetes; hence, the Nigerian estimate was extrapolated from pooled estimates in Cameroon, due to relatively similar geographic, ethnic, and socioeconomic patterns with Nigeria [4]. Without knowing estimates of the burden of diabetes, it will be difficult to put up a country-wide specific and appropriate public health and policy response to prevention, and management strategy to curb the increase. Hence, there is need for more research on the burden of diabetes in addition to its risk factors. We aimed to systematically review the literature on T2DM in Nigeria to provide national and regional estimates of the prevalence.

## Methodology

### Study area

**Fig. 1:**
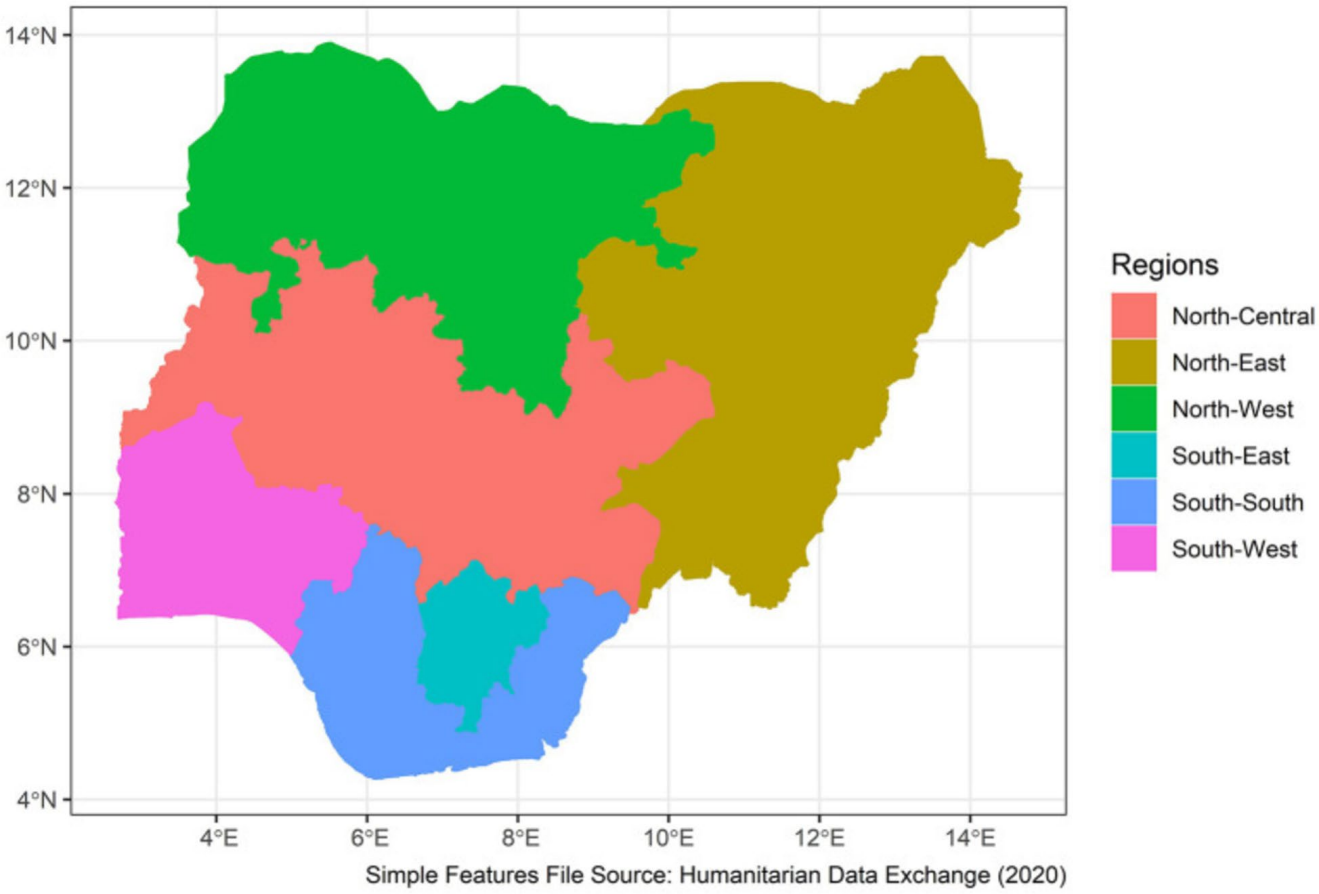
Shows the map of the geopolitical zones of the country

Nigeria is one of the nations located in the western part of Africa. It has an area of 910,770 sq km, and it is home to more than 250 ethnic groups [18]. It has 36 states (and a capital territory) which is grouped into 6 geo-political zones or regions. The current population of Nigeria is 228,973,667 (approximately 229 million) as of Saturday, June 2024, based on Worldometer estimate of the United Nations data [19]. This represents 2.78% of the total world population and ranks Nigeria as number 6th largest country by population in the world. A total of 43.4% of the population are under the age of 14 years. Overall, 53.9% of the population is between the ages of 15 and 64 years. Only 2.8% of the population are above the age of 65 years [20] Fig. 1.

### Study design

The present systematic review was conducted in April 2024 following the Preferred Reporting Items for Systematic Review and Meta-Analyses (PRISMA) guidelines [21, 22]. The search was performed after the protocol was accepted on the International Prospective Register of Systematic Reviews (PROSPERO) with the following identification number: CRD42024553891.

### Research question

The PICO framework was used for systematic reviews in line with the literature [23]. We aimed to assess data on the burden of T2DM and the risk factors for its occurrence.

Population (P): Individuals at least 18 years of age with T2DM

Intervention (I): Not applicable

Comparators (C): Not applicable

Outcomes (O): Diagnosis and risk of T2DM

Thus, our research questions were as follows:

- What is the prevalence of T2DM in Nigeria and its variations across geopolitical zones?
- What are the risk factors associated with the occurrence of T2DM in Nigeria?

The systematic review and meta-analysis were carried out using the Briggs Institute Reviewer manual [24] approach, which follows five steps: (i) defining the research question; (ii) finding pertinent studies; (iii) choosing studies; (iv) organizing the data; and (v) compiling, summarizing, and reporting the findings.

### Eligibility criteria

Studies were included in the review if they met the following criteria: (1) population based conducted among adults aged 18 years or more, residing in Nigeria, and reporting the prevalence, and/or risk or predisposing factors of T2DM in a Nigerian population, (2) hospital-based research with information on potential predisposing factors, (3). Studies in which the full text was published in English language (4) original research.

We excluded studies that were (1) primarily on type 1 diabetes; (2) conducted on paediatric population (0–17 years), or among populations of Nigerian origin residing outside Nigeria; (3) solely based on self-reported diagnosis of T2DM; (4) studies in which the data regarding T2DM were merged with data on different types of diabetes or studies in which the type of diabetes was not explicitly reported; (5) on diabetes but conducted among persons with co-morbidities; or (6). Studies such as RCTs, quasi-RCTs, crossover trials, controlled before and after studies, studies of simulation models, (7) case series, reviews, commentaries, experts’ opinion, conference proceedings, letters, or editorials.

### Data extraction

Literature search and assessment of eligible studies were conducted by two parallel reviewers, with an eligibility guideline to ensure that the selection criteria were consistently applied. Data on location, study period/publication year, study design, study setting (urban or rural), sample size, diagnostic criteria and mean age of the population were extracted. Extracted data were sorted by geopolitical zones in Nigeria and stored in Microsoft Excel file format for systematic review.

### Data analysis

We conducted a systematic synthesis to determine the prevalence and risk factors for the occurrence of T2DM in Nigeria. The Newcastle‒Ottawa scale was utilized by two unbiased reviewers to assess the quality of the selected papers [25]. The random-effects model was used for estimating the overall pooled prevalence of diabetes mellitus and its main components. A forest plot was also generated, and the effect size was used to assess the risk factors for T2DM. To determine the heterogeneity effect and assess the consistency of studies, we use I^2^ test statistics [26]. Consequently, since there was heterogeneity between the original studies (I^2^ = 97.1%, *p* < 0.001), a random effect model was needed. The potential sources of heterogeneity were examined using subgroup analysis and meta-regression. The publication bias was measured using the Deeks funnel plots and tests of Begg’s were used as suggested by different scholars [27]. All the statistical analyses were performed using STATA (version 16.0), Stata Corporation, Texas, USA. *p* < 0.05 was considered as statistically significant.

### Quality assessment

The following evaluation criteria were included in the Newcastle–Ottawa Scale (NOS), which was used to assess the quality of the studies: nonresponse rate, representativeness of the cases, adequate case definition, and identical ascertainment method. Supplementary File 1. The NOS's total score for quality assessment varied from 0 stars (lowest quality) to 9 stars (best quality). A study was often considered high-quality if it received seven or more stars. The selected articles ranged in quality from intermediate to high.

### Study selection

Duplicates were excluded by Mendeley (Elsevier, London, UK), using Rayyan software, two authors (KA) and (SA) independently screened the abstracts. A third author (MA) resolved any discrepancies between the two authors’ evaluations of the three sets of abstracts that were produced. The full texts of studies that could be relevant were assessed. Cohen's kappa coefficient was used to assess the agreement rate between the two reviewers throughout all the methodological phases, and the rate was 0.83, which is considered “strong agreement” [28].

## Results

### Characteristics of the selected studies

**Fig. 2:**
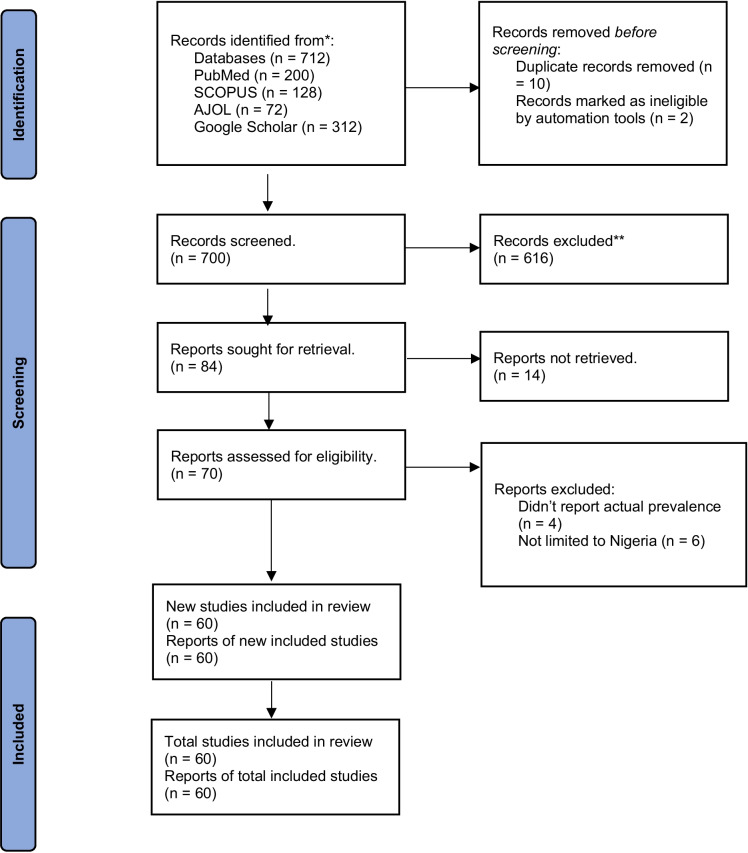
Flow diagram of the studies included in the meta-analysis

The literature search in the databases retrieved 712 titles. A total of 12 of these studies were found to be duplicated, and they were removed accordingly using Mendeley (Elsevier, London, UK), leaving 700 original studies. Next, the reviewers proceeded to analyze the 700 titles and abstracts, and 10 of these were excluded based on problems with reporting on the actual prevalence, and some studies included other neighbouring countries. In the next phase, 616 full texts were analyzed by the reviewers considering the inclusion and exclusion criteria for prevalence studies Fig. 2. Ultimately, 60 studies were included in the prevalence analysis Table S1.

### Description of the studies

**Fig. 3:**
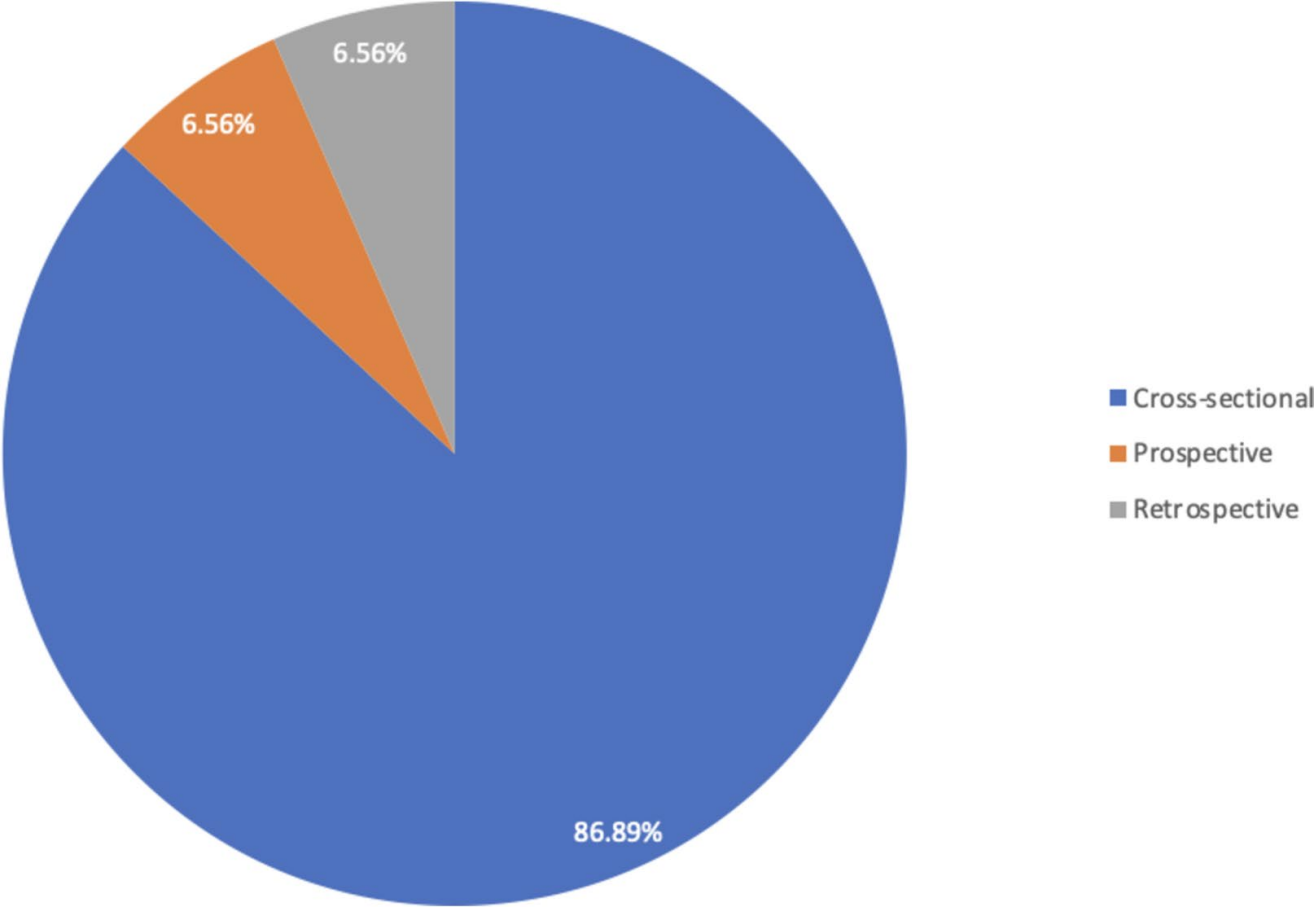
Distribution of the types of included studies

**Fig. 4:**
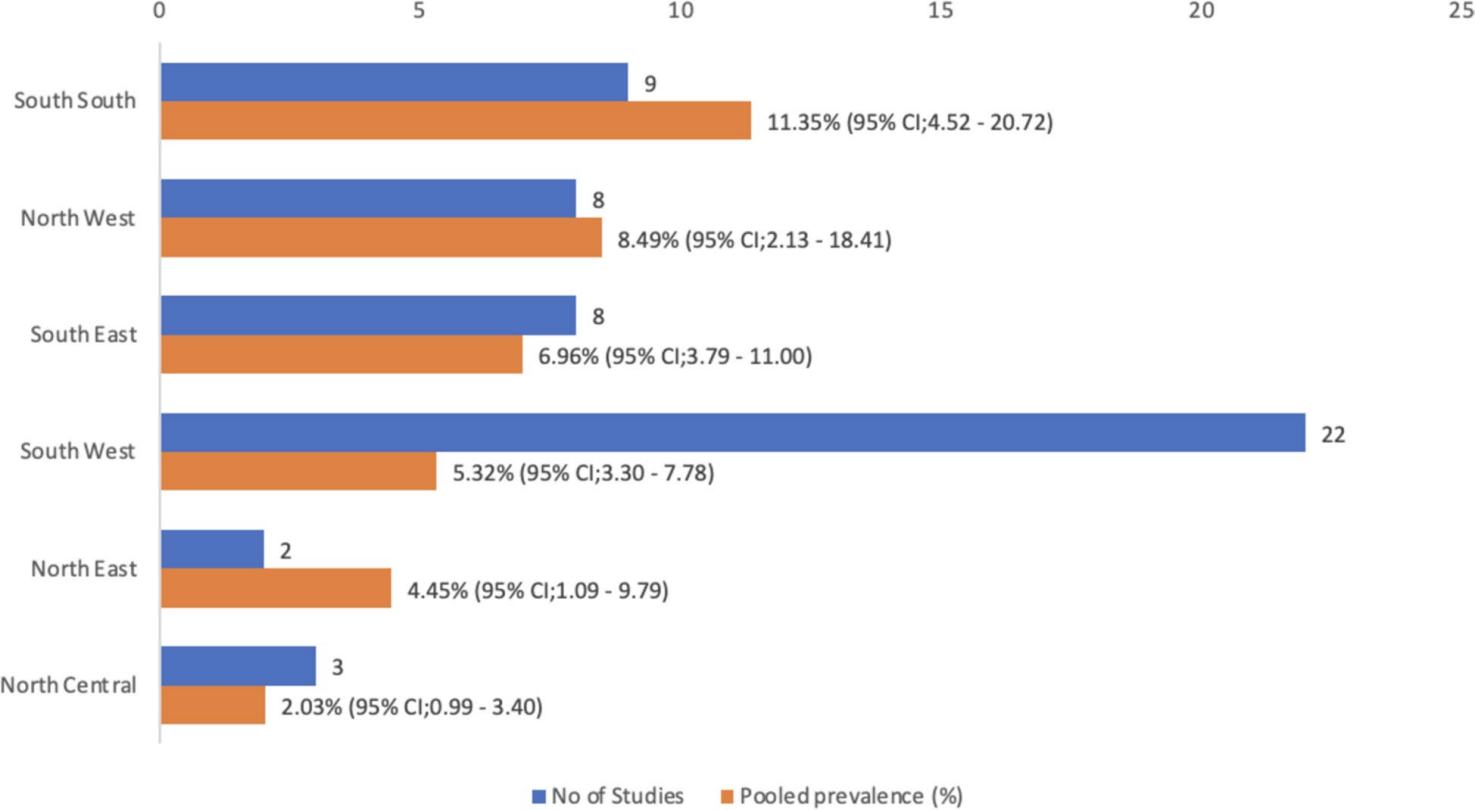
Number of studies used and pooled prevalence by geopolitical zone

Most studies were performed in a single state [19–23, 26–79], while two involved a different state [25, 80]; one study involved seven states [24], and most of the studies were performed in the Southwestern part of the country [19, 23, 25–27, 29, 40–42, 45, 48–51, 67–78]. The population included Doctors [19], adults [21, 22, 25, 42, 48–51, 64], and elderly individuals [23, 72]. Concerning the compositions of studies used, cross-sectional studies constituted 88.9% and both prospective and retrospective studies were 6.6% each Fig. 3. The sample sizes of the individual studies ranged between 60 and 58,567, with a total sample size of 124,876 patients. The study period ranged from 1989 to 2024, with a mean age of 48.9 years. Measurement bias was minimal as all the included studies used standard diagnostic criteria to ascertain the prevalence of diabetes. However, the funnel plot was asymmetrical, which is suggestive of publication bias across selected studies Fig. 4.

### Outcome measures

#### Prevalence rates of type 2 diabetes mellitus in Nigeria

**Fig. 5:**
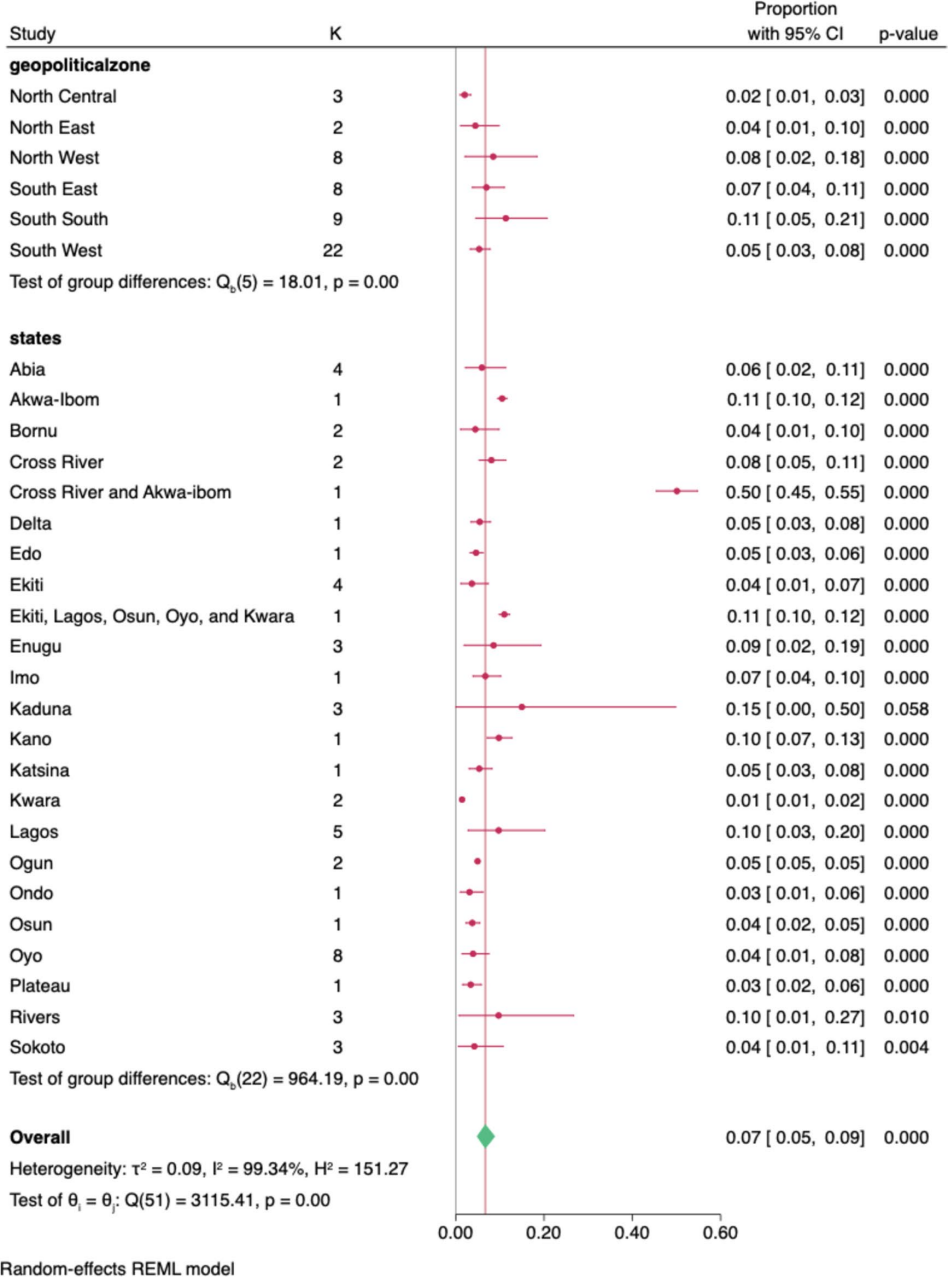
Forest plot showing prevalences of diabetes (overall, subregional and States

**Fig. 6:**
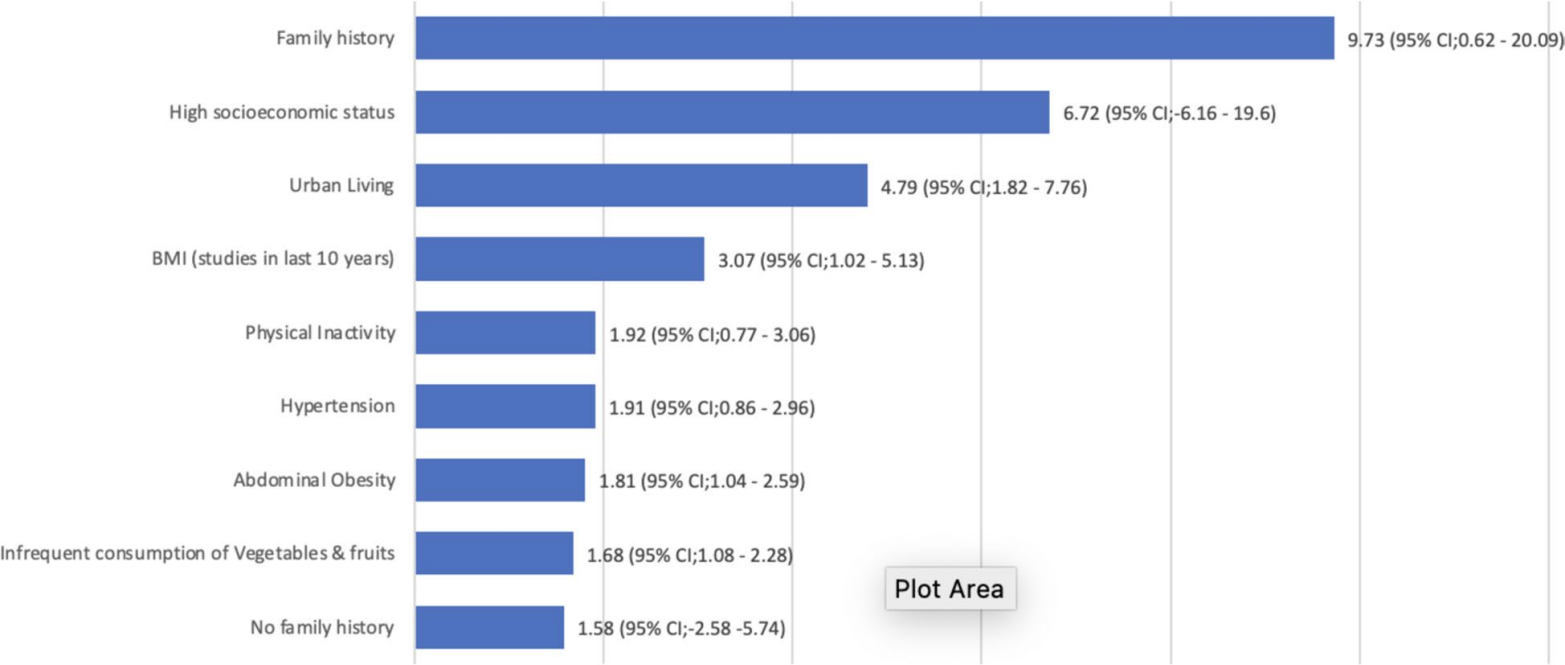
Pooled prevalence (Odd Ratios) of risk factors of T2DM

In this study, the pooled crude prevalence of type 2 diabetes in Nigeria was 7.0% (95% CI: 5.0%—9,0%), and the mean age (+ SD) of all the participants was 48.53 ± 11.21 years. In terms of the geographical distributions of type 2 diabetes occurrence, South‒South region of Nigeria had the highest pooled prevalence, 11.35%; (95%CI 4.52–20.72); then North‒West, 8.49%; (95%CI 2.13–5.18.41); 8.49%, and North Central had the lowest pooled prevalence at 2.03%. In the sub-analysis according to the States, Kaduna state had the highest pooled prevalence of T2DM, 2.01%; (95%CI 0.99–3.40), Akwa-Ibom, 11.0%, Kano, Lagos, and Rivers states had a pooled prevalence of 10% each, while Kwara state has the lowest prevalence of 1.20%, based on the number of studies published and selected per geopolitical zones Figs. 5 & 6.

#### Risk factors for type 2 diabetes

Risk factors for the pooled prevalence of T2DM were a family history of DM (9.73%; 95% CI 6.2–20.0); high socioeconomic status (6.72%; 95% CI6.16–19.6); urban living (4.79%; 95% CI 1,82–7.76); obesity (3.07%; 95% CI 1,02–5.13); physical inactivity (1.92%; 95% CI 1.77–3.06); hypertension (1.92%; 95% CI 1.86–2.96); abdominal obesity (1.81%; 95% CI 1.04–2.59); infrequent consumption of vegetables and fruits (1.68%; 95% CI 1.08–2.28); (8.0%; 95% CI 5.4–10.5).

#### Risk of publication bias

**Fig. 7:**
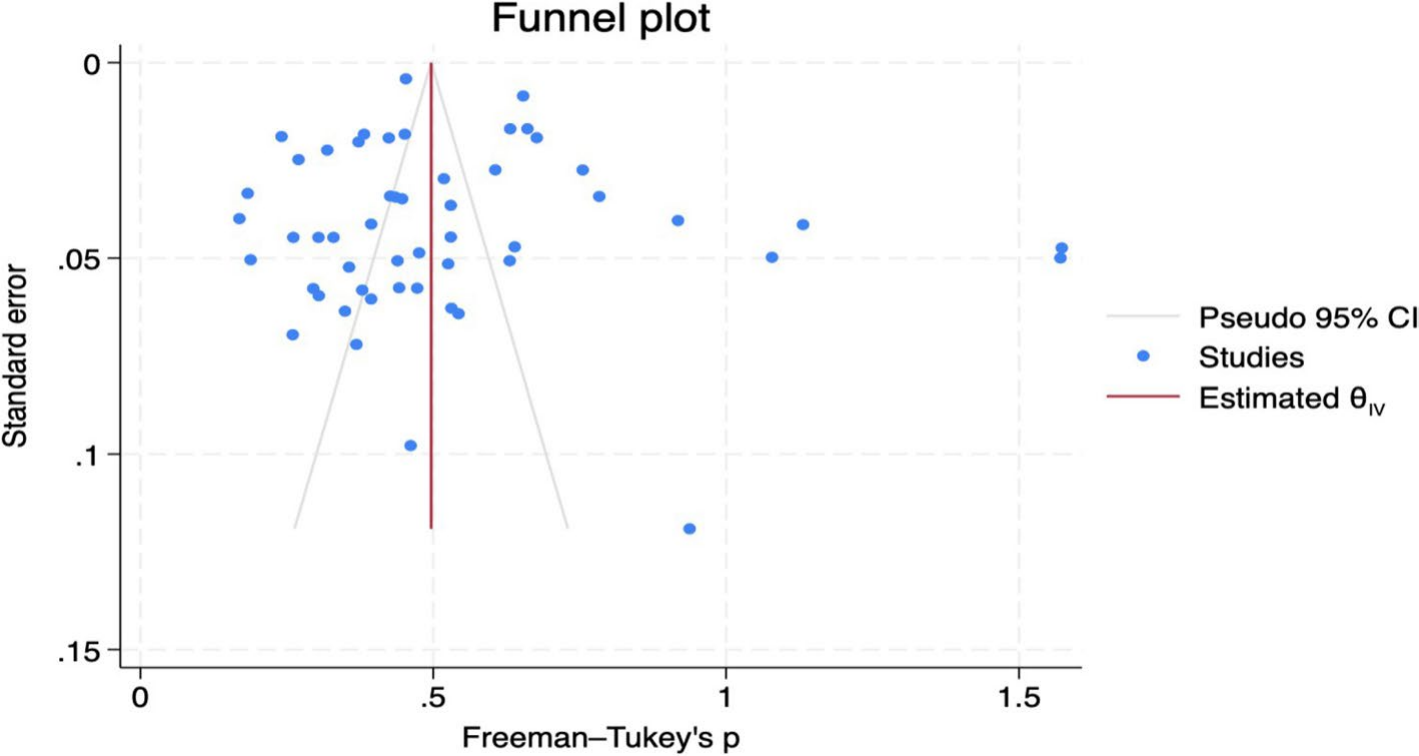
Forest plots for the prevalence of type 2 diabetes mellitus in Nigeria

This was determined by visual inspection of the funnel plot and using Egger’s test. The funnel plot showed asymmetrical, and most studies are outside of the Pseudo 95% confidence interval with the Egger’s test (*p* = 0.636); both the plot and the *p*-value show the existence of publication bias Fig. 7. This publication bias may be explained by the inclusion of articles with varied study designs, study population, period and setting and using different diagnosis methods.

## Discussion

These crude prevalence rates of diabetes in Nigeria were pooled from studies conducted between 1989 and 2024. The 2021 IDF estimate of 3.7% of diabetes prevalence in Nigeria was pooled from two studies conducted in neighboring nations of Cameroon and Ghana which are considered to share similar characteristics and predisposing factors for the occurrence of type 2 diabetes.in 2002 [79]. The pooled prevalence of type 2 diabetes obtained in this study showed that the IDF estimate did not truly represent the burden of diabetes in Nigeria as the prevalence was almost twice the estimate. The pooled T2DM prevalence of 7.0% obtained in this meta-analysis translates to 8.02 million Nigerians (1 in 14 adults) living with diabetes. Compared with the non-communicable disease (NCD) of 1992 population estimate of 2.2% by Akinkugbe et al. [5] and 5.77% pooled prevalence rates of diabetes obtained by Uloko et al. [7] in a similar meta-analysis in 2019, it showed that DM prevalence has increased by approximately 218.18% and 21.31% over a period of 32 and 6 years, respectively. This rising trend of diabetes burden is a global phenomenon including in Africa. It is observed in neighboring Cameroon where in 2003 the IDF reported an estimated 2.2% increase by more than three times to 7.1% in 2018 [81]. In the Eastern Mediterranean region where the IDF estimated prevalence of 6.8% in 2003 almost doubled to 12.2% in 2019 [82]. In Brazil where the IDF estimated prevalence of 6.8% in 2003 almost tripled to 18.5% in 2021 [83]. In England where the IDF estimated prevalence of 5.1% in 2003 increased almost seven times to 35.3% in 2011 [84]. In the United States where the IDF reported a prevalence of 8% in 2003 increased almost five times to 38% in 2011 [85].

This study findings also showed a regional variation in the pooled prevalence of diabetes, with the highest prevalence observed in the South-south geopolitical zone and the lowest rate in the North-central. This finding on the regional differences in the pooled prevalence of diabetes in Nigeria is similar to findings by Uloko et al. [7] except that the lowest pooled prevalence was found in the North-central as against the North-western region. This regional variation mirrors a similar finding for obesity and sedentary lifestyle which are major risk factors for T2DM [31].

Regarding potential risk factors for the occurrence of T2DM, we found positive family history of T2DM, age > 45 years, obesity including abdominal obesity, urban dwelling, infrequent intake of vegetables and high socioeconomic status as some of the significant risk factors responsible among Nigerians. According to our research findings, the single most important risk factor for T2DM in Nigeria is positive family history. These findings are consistent with those of InterAct studies [86, 87]. People who have a family history of diabetes are at least nine times more likely to have the disease compared to those without a family history. It is thought that shared environmental and genetic factors among family members contribute to this elevated risk. Family members' common eating and lifestyle choices can also lead to the development of obesity and sedentary behaviour, which increases the risk of diabetes [85–87].

It is generally acknowledged that having a body mass index (BMI) greater than 25 kg/m^2^ poses a serious risk of developing T2DM. Weight gain, especially abdominal obesity, is linked to insulin resistance, a condition in which cells lose their sensitivity to the physiological actions of insulin, resulting in hyperglycaemia. These individuals with obesity are also more likely to have hypertension and/or dyslipidaemia both of which are additional risk factors for diabetes. Type 2 diabetes is largely associated with age, with people 45 years of age and older having a heightened chance of developing the disease. As people age, their ability to use insulin gradually decreases, leading to a steady reduction in insulin sensitivity. Insulin resistance associated with aging, together with possible reductions in muscle mass and physical activity, can lead to hyperglycaemia [88–91]. For people older than 45 years, screening for diabetes is especially crucial since early identification and treatment can help avoid or delay diabetes-related complications. Adopting healthy lifestyle habits, including regular exercise, a balanced diet, and weight management, can significantly reduce the risk of developing T2DM in older adults.

Nigeria’s distinct socioeconomic and cultural setting necessitates a diversified strategy for controlling T2DM risk factors [92–94]. With the increasing rates of urbanization and sedentary lifestyles, it is imperative to address lifestyle factors, including nutrition and physical activity. Diabetes can be prevented in large part by encouraging public awareness campaigns about the value of healthy eating practices, decreased intake of processed foods, and increasing the consumption of fruits, vegetables, and whole grains. Promoting regular physical activity with programs such as walking and cycling infrastructure upgrades and community-based exercise regimens can help counteract the growing trend of sedentary behaviour that increases the risk of diabetes. Furthermore, for the early identification and treatment of diabetes risk factors in Nigeria, expanding access to screening programs and healthcare facilities is essential. Improving the infrastructure of primary healthcare and educating medical staff on how to offer complete lifestyle modifications.

### Strengths and limitations of review

The strength of this study is the large number of participants included in this systematic review of the literature and meta-analysis. Additionally, the included studies cover the whole six geopolitical of Nigeria, hence, enabling the determination of regional differences in the burden of diabetes. However, the studies used in the review were not evenly distributed across various parts of Nigeria as most studies selected were conducted in the Southern geopolitical zones of Nigeria, with the Northern zones having significantly fewer studies. Data retrieved from some studies were also incomplete, as results of some studies sampling strategy and study designs, were not always detailed. There were also sources of heterogeneity from study designs, measurement protocols, individual and population differences across selected studies. Also, our selection and quality criteria may have excluded low-quality studies, and we conducted subgroup meta-analyses on selected studies to identify other sources of heterogeneity that may further aid the interpretation of results. The results of sensitivity analysis and meta-regression in our study showed that the risk of bias might be the source of heterogeneity. Thus, researchers should pay attention to these issues to reduce the risk of bias of cross-sectional studies. In addition, we calculated ORs using raw (unadjusted) data from the studies rather than adjusting for potential underlying differences among study participants, the residual confounding was inevitable. However, non-significant findings from other meta-analyses between unadjusted associations and those adjusted for covariates suggest that this may not have a significant impact on the results [95]. The pooled estimate of this meta-analysis included studies that employed both fasting plasma glucose (FPG), casual/random plasma glucose, oral glucose tolerance test (OGTT) and glycated haemoglobin (HbA1c) in diagnosing diabetes. Although HbA1c is a sensitive marker for chronic hyperglycemia and useful in monitoring microvascular complications of diabetes [89], it is a less reliable tool for making a diagnosis of diabetes as its values can be affected by many factors causing both false positive or false negative results [94] Specifically, HbA1c values are known to be higher in individuals of African ancestry compared to Caucasians for the same level of plasma glucose levels in individuals with diabetes [94, 96–99]. Hence, this meta-analysis that included HbA1c-based studies might lead to an inflated pooled estimate of diabetes prevalence in Nigeria. A sensitivity analysis carried out by running the meta-analysis model with and without HbA1c-based studies showed that, in the context of this meta-analysis, including the HbA1c-based studies did not lead to an inflated prevalence of diabetes. Finally, some of the included studies are of low methodological quality with subjects used not representative of the general population or employing convenient sampling methods. This may have a negative effect on the validity of our estimates and makes the generalizability of the results challenging. However, the quality appraisal of the included studies might help in improving the validity as out of the 60 included studies only 12 were judged to be of good methodological quality and included in the quantitative analysis. The inclusion of studies that used cross-sectional design in this meta-analysis makes causal associations between diabetes and identified risk factors difficult. Also, our study findings revealed several possible risk factors associated with T2DM, which if controlled with useful preventive measures can help reduce the incidence of T2DM in Nigeria and by extension globally. However, since we included the cross-sectional studies, and extracted the characteristics at study level to explore the potential risk factors, the ecological fallacy may exist.

## Conclusion

The pooled prevalence of diabetes in Nigeria was found to be 7.0% (95% CI: 6.4–9.6%, *I*^2^ = 98.4%). According to the latest data by the United Nations, this translates to 8.02 million Nigerian adults with diabetes. This prevalence of diabetes in Nigeria was almost two times higher than the 3.6% estimate by the International Diabetes Federation in 2021. The pooled prevalence also represents an increase of almost 218% and 21.31% over a period of 32 and 6 years, respectively. Family history of diabetes, age > 45 years, obesity, infrequent intake of vegetables, and high socioeconomic status are significant risk factors for diabetes among Nigerians. Early identifications of these risks and lifestyle medication is recommended to reduce the increasing prevalence of this condition.

## Supplementary Information

Supplementary Material 1.

Supplementary Material 2.

## Data Availability

Data sharing is not applicable to this article as no datasets were generated or analysed during the current study.

## Abbreviations

BMI: Body mass index
DM: Diabetes mellitus
FPG: Fasting plasma glucose
HbA1c: Glycated haemoglobin
IDF: International Diabetes Federation
NCDs: Non-communicable diseases
OGTT: Oral glucose tolerance test
PRISMA: Preferred Reporting Items for Systematic Reviews and Meta-Analyses
RCT: Randomized controlled trial
T2DM: Type 2 diabetes mellitus
