# Supplementary material for: A systematic review and meta-analysis of the prevalence and risk factors of type 2 diabetes mellitus in Nigeria
Source: Clin Diabetes Endocrinol. 2024 Dec 6;10:43. doi: 10.1186/s40842-024-00209-1 (PMC11622640; doi:10.1186/s40842-024-00209-1)
Supplement: Supplementary file 1 — Supplementary Material 1. [file 40842_2024_209_MOESM1_ESM.docx]

Supplementary File 1. Newcastle-Ottawa Quality Assessment Form for Studies

| **Author/Year** | **Newcastle-Ottawa Scale** | | | | | | | | | | | |  | |
| --- | --- | --- | --- | --- | --- | --- | --- | --- | --- | --- | --- | --- | --- | --- |
|  | **Selection** | | | |  | **Comparability** | |  | **Outcome** | | | **Total** | **Quality** | |
|  | 1^a^ | 2^b^ | 3^c^ | 4^d^ |  | 5^e^ | 6^f^ |  | 7^g^ | 8^h^ | 9^i^ |  |  |  |
| Enikuomehin | ● | ● | ● | ● |  | - | - |  | ● | - | ● | 6 | Poor |  |
| Isa | ● | ● | ● | ● |  | ● | ● |  | ● | ● | ● | 9 | Good |  |
| Nyenwe | ● | ● | ● | ● |  | ● | ● |  | ● | - | ● | 8 | Good |  |
| Musa | ● | ● | ● | ● |  | ● | ● |  | ● | - | ● | 8 | Good |  |
| Balogun | ● | ● | ● | ● |  | ● | ● |  | ● | - | ● | 8 | Good |  |
| Ajayi | ● | ● | ● | ● |  | ● | ● |  | ● | - | ● | 8 | Good |  |
| Ibrahim | ● | ● | ● | ● |  | ● | ● |  | ● | - | ● | 8 | Good |  |
| Alebiosu | ● | ● | ● | ● |  | ● | ● |  | ● | - | ● | 8 | Good |  |
| Sabir | ● | ● | ● | ● |  | - | - |  | ● | - | ● | 6 | Poor |  |
| Oluwayemi | ● | ● | ● | ● |  | ● | ● |  | ● | ● | ● | 9 | Good |  |
| Erasmus | ● | ● | ● | ● |  | ● | ● |  | ● | - | ● | 8 | Good |  |
| Oghagbon | ● | ● | ● | ● |  | ● | ● |  | ● | - | ● | 8 | Good |  |
| Tagurum | ● | ● | ● | ● |  | ● | ● |  | ● | - | ● | 8 | Good |  |
| Gezawa | ● | ● | ● | ● |  | ● | ● |  | ● | - | ● | 8 | Good |  |
| Okesina | ● | ● | ● | ● |  | ● | ● |  | ● | - | ● | 8 | Good |  |
| Dahiru | ● | ● | ● | ● |  | ● | ● |  | ● | - | ● | 8 | Good |  |
| Sabir | ● | ● | ● | ● |  | - | - |  | ● | - | ● | 6 | Poor |  |
| Sabir | ● | ● | ● | ● |  | ● | ● |  | ● | ● | ● | 9 | Good |  |
| Sani | ● | ● | ● | ● |  | ● | ● |  | ● | - | ● | 8 | Good |  |
| Ijoma | ● | ● | ● | ● |  | ● | ● |  | ● | - | ● | 8 | Good |  |
| Akande | ● | ● | ● | ● |  | ● | ● |  | ● | - | ● | 8 | Good |  |
| Idowu | ● | ● | ● | ● |  | ● | ● |  | ● | - | ● | 8 | Good |  |
| Okurumeh | ● | ● | ● | ● |  | ● | ● |  | ● | - | ● | 8 | Good |  |
| Onyemelukwe | ● | ● | ● | ● |  | ● | ● |  | ● | - | ● | 8 | Good |  |
| Okon | ● | ● | ● | ● |  | - | - |  | ● | - | ● | 6 | Poor |  |
| Nalado | ● | ● | ● | ● |  | ● | ● |  | ● | ● | ● | 9 | Good |  |
| Amadi | ● | ● | ● | ● |  | ● | ● |  | ● | - | ● | 8 | Good |  |
| Okoduwa | ● | ● | ● | ● |  | ● | ● |  | ● | - | ● | 8 | Good |  |
| Nubila | ● | ● | ● | ● |  | ● | ● |  | ● | - | ● | 8 | Good |  |
| Omenai | ● | ● | ● | ● |  | ● | ● |  | ● | - | ● | 8 | Good |  |
| Owoaje | ● | ● | ● | ● |  | ● | ● |  | ● | - | ● | 8 | Good |  |
| Akande | ● | ● | ● | ● |  | ● | ● |  | ● | - | ● | 8 | Good |  |
| Kolawole | ● | ● | ● | ● |  | - | - |  | ● | - | ● | 6 | Poor |  |
| Aguocha | ● | ● | ● | ● |  | ● | ● |  | ● | ● | ● | 9 | Good |  |
| Ejike | ● | ● | ● | ● |  | ● | ● |  | ● | - | ● | 8 | Good |  |
| Ejim | ● | ● | ● | ● |  | ● | ● |  | ● | - | ● | 8 | Good |  |
| Ngwogu | ● | ● | ● | ● |  | ● | ● |  | ● | - | ● | 8 | Good |  |
| Nwatu | ● | ● | ● | ● |  | ● | ● |  | ● | - | ● | 8 | Good |  |
| Ogah | ● | ● | ● | ● |  | ● | ● |  | ● | - | ● | 8 | Good |  |
| Okpechi | ● | ● | ● | ● |  | ● | ● |  | ● | - | ● | 8 | Good |  |
| Osuji | ● | ● | ● | ● |  | - | - |  | ● | - | ● | 6 | Poor |  |
| Ekpenyong | ● | ● | ● | ● |  | ● | ● |  | ● | ● | ● | 9 | Good |  |
| Alikor | ● | ● | ● | ● |  | ● | ● |  | ● | - | ● | 8 | Good |  |
| Enang | ● | ● | ● | ● |  | ● | ● |  | ● | - | ● | 8 | Good |  |
| Isara | ● | ● | ● | ● |  | ● | ● |  | ● | - | ● | 8 | Good |  |
| Nwafor | ● | ● | ● | ● |  | ● | ● |  | ● | - | ● | 8 | Good |  |
| Oguoma | ● | ● | ● | ● |  | ● | ● |  | ● | - | ● | 8 | Good |  |
| Umoh | ● | ● | ● | ● |  | ● | ● |  | ● | - | ● | 8 | Good |  |
| Ogbera | ● | ● | ● | ● |  | ● | ● |  | ● | - | ● | 8 | Good |  |
| Akintunde | ● | ● | ● | ● |  | ● | ● |  | ● | - | ● | 8 | Good |  |
| Akinwale | ● | ● | ● | ● |  | ● | ● |  | ● | - | ● | 8 | Good |  |
| Ayodele | ● | ● | ● | ● |  | - | - |  | ● | - | ● | 6 | Poor |  |
| Ezenwaka | ● | ● | ● | ● |  | ● | ● |  | ● | ● | ● | 9 | Good |  |
| Ogunmola | ● | ● | ● | ● |  | ● | ● |  | ● | - | ● | 8 | Good |  |
| Ohwovoriole | ● | ● | ● | ● |  | ● | ● |  | ● | - | ● | 8 | Good |  |
| Ojewale | ● | ● | ● | ● |  | ● | ● |  | ● | - | ● | 8 | Good |  |
| Oladapo | ● | ● | ● | ● |  | ● | ● |  | ● | - | ● | 8 | Good |  |
| Oluyombo | ● | ● | ● | ● |  | ● | ● |  | ● | - | ● | 8 | Good |  |
| Olatunbusun | ● | ● | ● | ● |  | ● | ● |  | ● | - | ● | 8 | Good |  |
|  |  |  |  |  |  |  |  |  |  |  |  |  |  |  |

^a^ Representativeness of the exposed cohort

^b^ Selection of the non-exposed cohort

^c^ Ascertainment of exposure

^d^ Demonstration that outcome of interest was not present at start of study

^e^ Comparability of cohorts on the basis of the design or analysis (adjusted for age)

^f^ Comparability of cohorts on the basis of the design or analysis (adjusted for any other factor)

^g^ Assessment of outcome

^h^ Was follow-up long enough for outcomes to occur

^i^ Adequacy of follow-up of cohorts
