# Supplementary material for: A systematic review and meta-analysis of the prevalence and risk factors of type 2 diabetes mellitus in Nigeria
Source: Clin Diabetes Endocrinol. 2024 Dec 6;10:43. doi: 10.1186/s40842-024-00209-1 (PMC11622640; doi:10.1186/s40842-024-00209-1)
Supplement: Supplementary file 2 — Supplementary Material 2. [file 40842_2024_209_MOESM2_ESM.docx]

Table 1: Characteristics of selected studies on T2DM in Nigeria

| Author | Ye ar | State (s) | Geopolit ical Zone | Popul ation Group | Ty pe s of stu dy | Samp le Size | Prevale nce | Ma le to Fe ma le rati  o | Mean age | Risk Factors |
| --- | --- | --- | --- | --- | --- | --- | --- | --- | --- | --- |
| Enikuome | 20 | Ondo | Southwe | Doctors | Cross- | 192 | 3.1% | 110:82 | NR | Age ≥ 45 years |
| hin [29] | 21 |  | st |  | sectional |  | (6) |  |  | (AOR:9.08; CI |
|  |  |  |  |  |  |  |  |  |  | 3.13- |
|  |  |  |  |  |  |  |  |  |  | 26.33; p = |
|  |  |  |  |  |  |  |  |  |  | <0.001) |
|  |  |  |  |  |  |  |  |  |  | Family |
|  |  |  |  |  |  |  |  |  |  | history of |
|  |  |  |  |  |  |  |  |  |  | DM |
|  |  |  |  |  |  |  |  |  |  | (AOR:9.93; |
|  |  |  |  |  |  |  |  |  |  | CI:3.25- |
|  |  |  |  |  |  |  |  |  |  | 30.39; p = |
|  |  |  |  |  |  |  |  |  |  | <0.001); |
|  |  |  |  |  |  |  |  |  |  | Abdominal |
|  |  |  |  |  |  |  |  |  |  | obesity |
|  |  |  |  |  |  |  |  |  |  | (AOR:6.66; |
|  |  |  |  |  |  |  |  |  |  | CI:2.08- 21.29; |
|  |  |  |  |  |  |  |  |  |  | p= < 0.001); and |
|  |  |  |  |  |  |  |  |  |  | Infrequent |
|  |  |  |  |  |  |  |  |  |  | Dietary |

|  |  |  |  |  |  |  |  |  |  | intake of fruits and vegetable  (AOR:3.11;CI:1. 03:9.37: p =  0.04).  Male sex |
| --- | --- | --- | --- | --- | --- | --- | --- | --- | --- | --- |
| Isa [30] | 20  16 | Plate  au | North  Central | HIV  patients | Cohort | 2632 | NR | 924:17  08 | 37.4 ± 9.7 | NR |
| Nyenwe [31] | 20 | River | South‒ | Adults | Cross- | 502 | 6.8 | 273:22 | 48 ± 9.2 | Body mass index |
|  | 03 | s | south 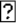 |  | sectional |  | % | 9 |  | (BMI) |
|  |  |  |  |  |  |  | (CI |  |  | > or = 25 kg/m2 |
|  |  |  |  |  |  |  | =4. |  |  | WHR > or = |
|  |  |  |  |  |  |  | 6- |  |  | 0.85, family |
|  |  |  |  |  |  |  | 9.0 |  |  | history of |
|  |  |  |  |  |  |  | %) |  |  | diabetes, |
|  |  |  |  |  |  |  |  |  |  | Physical |
|  |  |  |  |  |  |  |  |  |  | inactivity, |
|  |  |  |  |  |  |  |  |  |  | Heavy |
|  |  |  |  |  |  |  |  |  |  | consumption of |
|  |  |  |  |  |  |  |  |  |  | alcohol, older |
|  |  |  |  |  |  |  |  |  |  | age as well as |
|  |  |  |  |  |  |  |  |  |  | high social |
|  |  |  |  |  |  |  |  |  |  | status and |
|  |  |  |  |  |  |  |  |  |  | Hausa- |
|  |  |  |  |  |  |  |  |  |  | Fulani or Ibibio |

|  |  |  |  |  |  |  |  |  |  | Origin |
| --- | --- | --- | --- | --- | --- | --- | --- | --- | --- | --- |
| Musa [32] | 20  22 | Kogi | North  Central | Adolesce  nts | Cross-  sectional | 403 | NR | 202:20  1 | 14.7 ±2.3 | NR |
| Balogun [33] | 20  12 | Oyo | Southwe st | Elderly | Cohort | 1330 | 8.87% (CI 6.45-  12.19) | 615:71  5 | 77.3± 0.3 | Urban living High socioecono mic factor  (AOR:3.00) |
| Ajayi [75] | 20  23 | Eki ti, La gos  ,  Os un, Oy o, and Kw ara | Southwe st | Adults | Cross- sectional | 2708 | 10.7% | 925:17  59 | 48.1 ±  15.8 | A  g e G  e n d e r  Level of education Occupatio n  Marital status Elevated Blood pressure (OR:1.33) |

| Ibrahim [34] | 20  24 | Ekiti | Southwe st | NR | Cross- sectional | 280 | NR | 105:17  5 | 54.5 ±  11.4 | NR |
| --- | --- | --- | --- | --- | --- | --- | --- | --- | --- | --- |
| Alebiosu [35] | 20  13 | Ogun | Southwe st | NT | Longitu dinal observat ional | 58,56  7 | 5.05%  (2,956) | 23,667:  34,990 | 40.69 ±  16.27 | Systemic hypertension (AOR: 0.45) BMI  Waist  Circumference |
| Sabir [36] | 20  17 | Soko to | Northwe st | Fulani | Cross- sectional | 389 | 16.9%  (37) | 199:19  0 | 39.3 ±  14.2 | Mal e sex (AOR: 1.1, P <0.001, CI: 3.9-6.4)), Incr ease age Hig h BlM I  High Waist- Circumference ratio Systemic  hypertension |
| Oluw ayemi [37] | 20  15 | Ekiti | Southwe st | Adolesce nts | Cross- sectional | 628 | 0.6%(4) | 346:  282 | 14.2 ±  1.7 | Female gender (AOR:1.70. P<0.001) Early adolescent s (AOR:1.39, P<0.001) Family history of  Obesity (p<0.045) |

| Erasmus [38] | 19  89 | Kwar a | Northcen tral | NR | Cross- sectional | 2800 | 1.43%  (40) | 1727:  1073 | 60.7 | Incr ease Age Obe sity  Chronic Alcohol  intake |
| --- | --- | --- | --- | --- | --- | --- | --- | --- | --- | --- |
| Ogha gbon [39] | 20  08 | Kwar a | North Central | Workers | Cross- sectional | 281 | 1.5% | 211: 70 | 50.5 | NR |
| Tagurum  [40] | 20  14 | Plate au | Northcen tral | Adults | Cross- sectional | 295 | 5.1%  (10) | 43:152 | 47.5±18.  8 | Consumptio n of fast food (p = 0.01)  Inadequate physical activity  High salt intake (AOR: 0.57; P=0.07)  History in the siblings Age >  45 (AOR:1.64; P= 0.38) |
| Gezawa [41] | 20  09 | Born u | North East | General | Cross- sectional | 242 | 7.0% | 96:146 | 39.4±  13.7 | Age > 45  BMI >/= 25 (male AOR: 1.65; 95%CI=0.75-3.60; P=0.036)  (female: AOR:1.63; 95%CI=0.56-4.78; P=0.025) Waist-hip ratio: 0.85 Waist circumferen ce Family  History |

| Okesina  [42] | 19  99 | Born u | Northeas t | NR | Cross- sectional | 500 | 2.6% | 278:  222 | 45.5 | NR |
| --- | --- | --- | --- | --- | --- | --- | --- | --- | --- | --- |
| Dahiru  [43] | 20  07 | Kadu na | Northwe st | NR | Cross sectional | 299 | 2.0% | 94: 105 | 59.4 | NR |
| Sabir [44] | 20  11 | Soko to | Northwe st | Fulanis | Cross- sectional | 389 | 4.6% | 199:19  0 | 39.3±14.  2 | Central obesity BMI >30  Overweig ht (p < 0.05)  Smoking Alcohol intake Family  History |
| Sabir [10] | 20  13 | Soko to | Northwe st | Fulanis | Cross- sectional | 393 | 0.8% | 210:  183 | 44.8 ±  13.6 | Central obesity BMI >30  Overweig ht (p < 0.05)  Smoking Alcohol intake Family  History |
| Sani [45] | 20  10 | Katsi na | North West | Adult | Cross- sectional | 300 | 5.3% | 129:  171 | 37.6±10.  6 | Waist circumference, Total cholesterol, Alcohol intake (AOR: 0.27, AOR: 0.46; AOR: 0.06 P <0.05) |

| Ijoma [46] | 20  19 | Enug u | Southeas t | NR | Cross- sectional | 613 | 19.6% | NR | NR | NR |
| --- | --- | --- | --- | --- | --- | --- | --- | --- | --- | --- |
| Akande [47] | 20  13 | Oyo | Southwe st | Adult | Cross- sectional | 70 | 20% | NR | 55.8±10.  3 | Hyperte nsion BMI |

| Idowu [48] | 20  22 | Ogun | Southwe st | Adult | Cross- sectional | 273 | 3.8% | NR | 54.20±16  .61 | NR |
| --- | --- | --- | --- | --- | --- | --- | --- | --- | --- | --- |
| Okuru meh [49] | 202  2 | Ekiti | Southwe st | NR | Cross- sectional | 126 | NR | 56:70 | 57.8 ±  12.9 | N R |
| Onyemel ukwe [50] | 202  0 | Kadu na | Northwe st | Health care worke rs | Cross- sectional | 377 | 6.5% | NR | NR | N R |
| Okon [51] | 200  8 | Cr os s Ri ve r an d Ak wa  -  ib o  m | South South | ABO/Rh esus blood groups | Cross- sectional | 445 | 50.1% | NR | 50.9 ±  11.4 | N R |
| Nalado [52] | 201  5 | Kano | North  West | NR | Cross-  sectional | 450 | 9.78% | 171:  279 | 40.27 ±  16.41 | N  R |

|  |  |  |  |  |  |  |  |  |  |  |
| --- | --- | --- | --- | --- | --- | --- | --- | --- | --- | --- |
| Amadi [53] | 202  2 | Lago s | Southwe st | Hyperten sive patients | Cross- sectional | 582 | 28.7% | 309:27  3 | 56.2  ±13.6 | N R |
| Okoduwa  [54] | 201  4 | Kadu na | Northwe st | NR | Cross- sectional | 400 | 50% | 192:20  8 | NR | N R |
| Omenai [55] | 202  0 | Oyo | Southwe st | Autop sy case | Cross- sectional | 1092 | NR | 437:  873 | NR | N R |
| Owoaje [56] | 199  7 | Oyo | Southwe  st | Adult | Cross-  sectional | 247 | 2.8% | NR | NR | N  R |

|  |  |  |  |  |  |  |  |  |  |  |
| --- | --- | --- | --- | --- | --- | --- | --- | --- | --- | --- |
| Akande [57] | 20  13 | Oyo | Southwe st | Adults with essential hyperten  sion | Cross- sectional | NR | NR | NR | NR | Hypertension (AOR: 2.915; 95% CI: 1.526-5.056)  Waist circumference (AOR: 1.050; 95%CI: 1.010-1.090) (p=0.288) |

| Kolawole  [58] | 20  00 | Oyo | Southwe st | Adult | Prospecti ve cohort | 105 | NR | 62:43 | NR | NR |
| --- | --- | --- | --- | --- | --- | --- | --- | --- | --- | --- |
| Aguocha  [59] | 20  13 | Abia | Southeas t | NR | Retrospe ctive cross- sectional | 1124 | NR | NR | 55±16 | NR |
| Ejike [60] | 20  15 | Abia | Southeas t | Adult | Cross- sectional | 365 | 3.0% | 172:19  3 | 46 | BMI (AOR: 0.282; p ,0.001)  WHR (0.317; p<0.001) |
| Ejim [61] | 20  11 | Enug u | Southeas t | Middle- aged and elderly | Cross- sectional | 858 | 4.43% | 247:61  1 | 59.8 | BMI  Hypertensio n Abdominal  Obesity |
| Ngwogu [62] | 20  12 | Abia | Southeas t | NR | Cross- sectional | 853 | 14.55% | 326:52  7 | 56.4±12.  7 | NR |
| Nwatu [63] | 20  15 | Enug u | Southeas t | NR | Cross- sectional | 824 | 4.8% | 286:53  8 | 51.1±16.  2 | Age  > 45  BM I  >/= 25 (AOR: 0.06)  Hypertension (AOR: 0.83)  High total  Cholesterol  Central obesity (AOR:0.186) |

| Ogah [64] | 20  13 | Abia | Southeas t | Adult | Cross- sectional | 2983 | 5% | 1430:1  553 | 41.7±18.  5 | NR |
| --- | --- | --- | --- | --- | --- | --- | --- | --- | --- | --- |
| Okpechi [65] | 20  13 | Abia | South East | NR | Cross- sectional | 2983 | 3.6% | 1430:1  553 | 41.7±0.3 | NR |
| Osuji [66] | 20  12 | Imo | South East | Women | Cross- sectional | 253 | 6.7% | NR | 53.4 | NR |
| Ekpen yong  [67] | 20  12 | Ak wa  -  Ib om | South south | Adult | Cross sectional | 3500 | 10.51% | 1532:  1968 | 49.8±0.3  4 | Age  > 45 (AOR: 0.83; p<0.005)  Obe sity Waist  circumference WHR  Dietary intake Physical Inactivity Smoking  Family history |
| Alikor [68] | 20  15 | River s | South‒ south 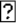 | NR | Cross- sectional | 500 | 2.2% | 156:34  4 | 41.32±17 | NR |

| Enang  [69] | 20  14 | Cr os s Ri ve  rs | South‒ south 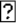 | NR | Cross- sectional | 1134 | 6.5% | 645:49  8 | 38.9  ±11.1 | NR |
| --- | --- | --- | --- | --- | --- | --- | --- | --- | --- | --- |
| Isara | 20 | Edo | South‒ | NR | Cross- | 845 | 4.6% | 263:58 | 56.4 ± | A |
| [70] | 15 |  | south 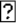 |  | sectional |  |  | 2 | 16.3 | ge |
|  |  |  |  |  |  |  |  |  |  | Ge |
|  |  |  |  |  |  |  |  |  |  | nd |
|  |  |  |  |  |  |  |  |  |  | er |
|  |  |  |  |  |  |  |  |  |  | B |
|  |  |  |  |  |  |  |  |  |  | M |
|  |  |  |  |  |  |  |  |  |  | I |
|  |  |  |  |  |  |  |  |  |  | >/ |
|  |  |  |  |  |  |  |  |  |  | = |
|  |  |  |  |  |  |  |  |  |  | 25 |
| Nwafor [71] | 200  1 | River s | South South | NR | Cross- sectional | 403 | 26.3% | 223:18  0 | 61.5 | N R |
| Oguoma | 20 | Delta | South‒ | Adult | Cross- | 422 | 5.4%(9 | 149:27 | 40.6± | Urban living  Total cholesterol (p=0.001; AOR; 0.02)  Total triacylglycerol (p=0.005)  BMI (p=0.03; AOR:1.09 |
| [72] | 15 |  | south 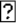 |  | sectional |  | 5% | 3 | 20.7 |  |
|  |  |  |  |  |  |  | CI: 3.2- |  |  |  |
|  |  |  |  |  |  |  | 7.6%) |  |  |  |
| Umoh [73] | 201  2 | Cross River | South‒ south 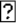 | NR | Cross- sectional | 3490 | 9.64% | 2202:1  288 | 45.8 ±  12.9 | N R |
| Ogbera [74] | 200 | Lago | Southwe | NR | Retrospec | 13,79 | 10.3% | NR | NR | N |

|  | 7 | s | st |  | tive | 7 |  |  |  | R |
| --- | --- | --- | --- | --- | --- | --- | --- | --- | --- | --- |
| Ajayi [75] | 200  9 | Ekiti | Southwe st | NR | Retrospec tive | 2,696 | 4.4% | 1509:  1187 | 57 ± 16.2 | N R |
| Ogbera [76] | 200  7 | Lago s | Southwe st | NR | Retrospec tive | 1327 | 13.6% | NR | NR | N R |
| Akint unde [77] | 201  4 | Oyo | Southwe st | Universit y staff | Cross- sectional | 206 | 1.5% | 96:110 | 45.3 ±  7.9 | N R |
| Akinwale  [78] | 201  3 | Lago s | Southwe st | NR | Cross- sectional | 2434 | 3.4% | 1152:1  282 | NR | N R |
| Ayodele [79] | 200  9 | Osun | Southwe st | NR | Cross- sectional | 586 | 3.8% | 360:22  6 | 42.4 ±  11.2 | N R |

| Ezenwaka [99] | 199  7 | Oyo | Southwe st | Elderly | Cross- sectional | 500 | 1.6% | 295:20  5 | 60.8 | NR |
| --- | --- | --- | --- | --- | --- | --- | --- | --- | --- | --- |
| Ogunmola [100] | 201  3 | Ekiti | Southwe st | NR | Cross- sectional | 104 | 4.8% | 33:71 | 66.77 ±  12.06 | N R |
| Ohwovori ole [101] | 198  8 | Lago s | Southwe st | NR | Cross- sectional | 1627 | 1.8% | 1050:5  77 | 44.2 | N R |
| \| Ojewale [102] \| \| --- \| | 201  2 | Oyo | Southwe st | NR | Cross- sectional | 301 | 4.7% | 112:18  9 | 49±6.47 | N R |
| Oladapo [103] | 201  0 | Oyo | Southwe st | Yoruba | Cross- sectional | 2000 | 2.5% | 873:11  27 | 42.1 ±  21.6 | N R |
| Oluyo mbo [104] | 201  5 | Ekiti | Southwe st | NR | Cross- sectional | 750 | 6.8% | 221:52  9 | 61.7±18.  2 | N R |
| Olatunbus un [105] | 199  5 | Oyo | Southwe st | NR | Cross- sectional | 894 | 0.8% | NR | 40.8% | N R |

NR: Not reported
